# Bis(3-aza­niumylprop­yl)aza­nium hexa­chlorido­bis­muthate(III) monohydrate

**DOI:** 10.1107/S1600536813030900

**Published:** 2013-11-16

**Authors:** Nizar Elfaleh, Hassen Chouaib, Slaheddine Kamoun

**Affiliations:** a Laboratoire de Génie des Matériaux et Environnement (LR11ES46), BP 1173, ENIS, Sfax, Tunisia

## Abstract

The asymmetric unit of the title compound, (C_6_H_20_N_3_)[BiCl_6_]·H_2_O, consists of a triprotonated bis­(3-aza­niumylprop­yl)aza­nium cation, two halves of an octahedral [BiCl_6_]^3−^ anion, each of the Bi^III^ atoms lying on an inversion centre, and a water mol­ecule. In the crystal, the anions and water mol­ecules are linked by O—H⋯Cl hydrogen bonds, forming chains running parallel to [0-11]. The anionic chains and the cations are further linked into a three-dimensional network by N—H⋯Cl and N—H⋯O hydrogen-bond inter­actions.

## Related literature

For related structures, see: Chaabouni *et al.* (1998 ▶); Fu *et al.* (2005 ▶); Rhandour *et al.* (2011 ▶); Ouasri *et al.* (2013 ▶). For bond-valence-sum calculations, see: Brown & Altermatt (1985 ▶). For van der Waals radii, see: Pauling (1960 ▶).
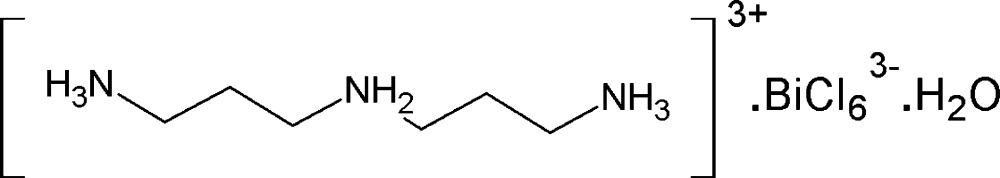


## Experimental

### 

#### Crystal data

(C_6_H_20_N_3_)[BiCl_6_]·H_2_O
*M* *_r_* = 573.95
Triclinic, 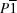

*a* = 7.6891 (1) Å
*b* = 10.8642 (1) Å
*c* = 11.9867 (1) Å
α = 93.349 (1)°
β = 108.509 (1)°
γ = 109.387 (1)°
*V* = 880.54 (2) Å^3^
*Z* = 2
Mo *K*α radiation
μ = 10.91 mm^−1^
*T* = 296 K
0.1 × 0.1 × 0.1 mm

#### Data collection

Bruker APEXII CCD diffractometer
Absorption correction: multi-scan (*SADABS*; Bruker, 2006 ▶) *T*_min_ = 0.336, *T*_max_ = 0.349
11734 measured reflections
5325 independent reflections
4009 reflections with *I* > 2σ(*I*)
*R*_int_ = 0.025

#### Refinement

*R*[*F* ^2^ > 2σ(*F* ^2^)] = 0.021
*wR*(*F* ^2^) = 0.057
*S* = 0.92
5325 reflections
168 parameters
3 restraints
H atoms treated by a mixture of independent and constrained refinement
Δρ_max_ = 1.07 e Å^−3^
Δρ_min_ = −0.90 e Å^−3^

### 

Data collection: *APEX2* (Bruker, 2006 ▶); cell refinement: *SAINT* (Bruker, 2006 ▶); data reduction: *SAINT*; program(s) used to solve structure: *SHELXS97* (Sheldrick, 2008 ▶); program(s) used to refine structure: *SHELXL97* (Sheldrick, 2008 ▶); molecular graphics: *Mercury* (Macrae *et al.*, 2008 ▶); software used to prepare material for publication: *WinGX* (Farrugia, 2012 ▶) and *publCIF* (Westrip, 2010 ▶).

## Supplementary Material

Crystal structure: contains datablock(s) I, New_Global_Publ_Block. DOI: 10.1107/S1600536813030900/rz5091sup1.cif

Structure factors: contains datablock(s) I. DOI: 10.1107/S1600536813030900/rz5091Isup2.hkl

Supplementary material file. DOI: 10.1107/S1600536813030900/rz5091Isup3.mol

Additional supplementary materials:  crystallographic information; 3D view; checkCIF report

## Figures and Tables

**Table 1:** Hydrogen-bond geometry (Å, °)

*D*—H⋯*A*	*D*—H	H⋯*A*	*D*⋯*A*	*D*—H⋯*A*
N1—H1*A*⋯Cl4^i^	0.89	2.34	3.174 (3)	156
N1—H1*B*⋯Cl2^ii^	0.89	2.73	3.339 (3)	127
N1—H1*B*⋯Cl1^iii^	0.89	2.82	3.474 (3)	132
N1—H1*C*⋯Cl3^ii^	0.89	2.43	3.293 (3)	163
N2—H2*B*⋯O*W*	0.90	1.91	2.804 (4)	173
N2—H2*A*⋯Cl2^iii^	0.90	2.63	3.316 (3)	134
N2—H2*A*⋯Cl1^ii^	0.90	2.71	3.347 (3)	129
N3—H3*A*⋯Cl6	0.89	2.48	3.362 (3)	169
N3—H3*B*⋯Cl1^iv^	0.89	2.81	3.412 (3)	126
N3—H3*B*⋯Cl3^v^	0.89	2.81	3.612 (3)	151
N3—H3*C*⋯Cl5^vi^	0.89	2.44	3.269 (3)	155
O*W*—H1*W*⋯Cl6	0.84 (2)	2.83 (3)	3.620 (4)	157 (6)
O*W*—H2*W*⋯Cl3^ii^	0.86 (2)	2.82 (4)	3.478 (4)	135 (5)

Symmetry codes: (i) 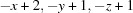; (ii) 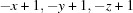; (iii) 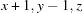; (iv) 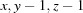; (v) 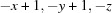; (vi) 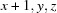.

**Fig. 1.**
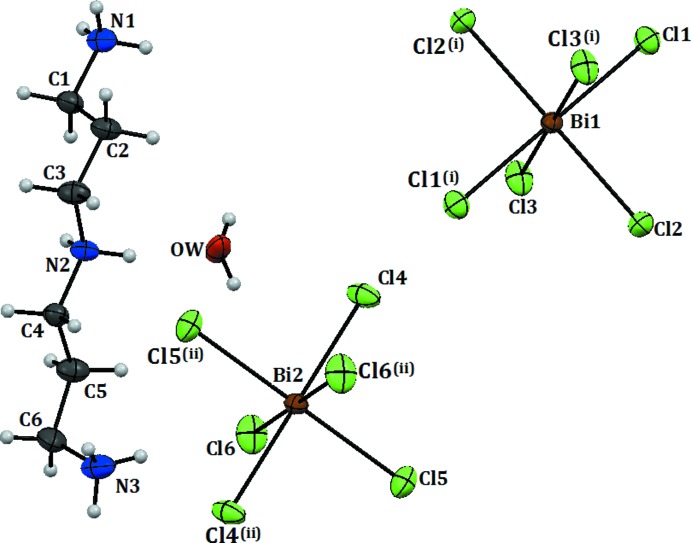
A view of the asymmetric unit of the title compound with displacement ellipsoids drawn at the 50% probability level. Symmetry codes: i -x+1,-y+2,-z+1; (ii) -x+1,-y+1,-z.

**Fig. 2.**
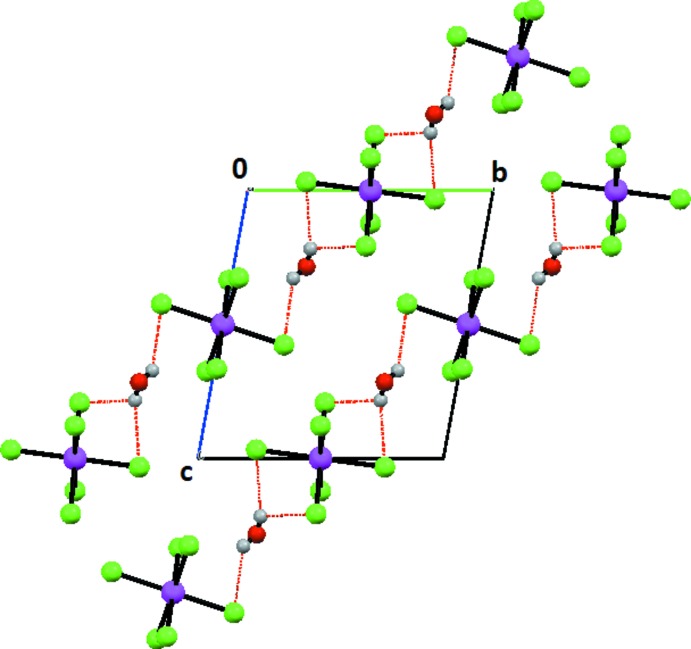
Arrangement of the anionic chains viewed along the a axis (purple= bismuth, green = chloride, red = oxygen, grey = hydrogen). Intermolecular hydrogen bonding is shown as red dashed lines.

**Fig. 3.**
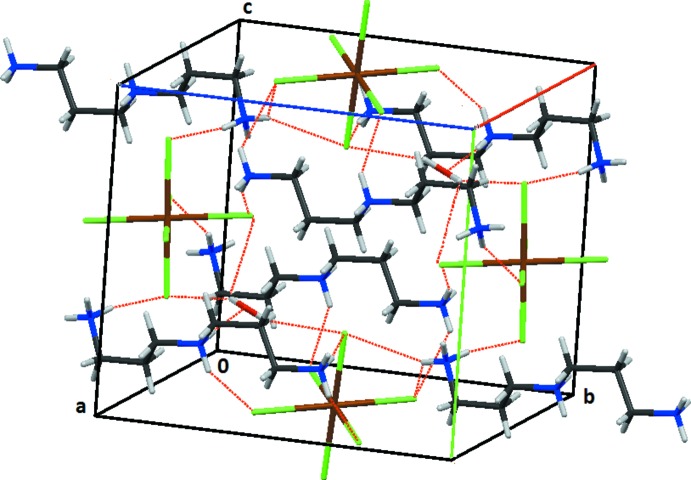
Crystal packing of the title compound showing the hydrogen bonding network as red dashed lines.

## supplementary crystallographic information

### 1. Comment

This work is a part of our study on the crystal structure of alkylpolyammoniumbismuthate(III) chorides. This investigation was extended to aliphatic diamines of general formula NH_2_(CH_2_)_n_NH_2_ (Chaabouni *et al.*, 1998; Rhandour *et al.*, 2011; Ouasri *et al.*, 2013) and triamines of general formula NH_2_(CH_2_)_n_NH(CH_2_)_n_NH_2_ (Fu *et al.*, 2005) in order to examine the effect of the flexible cation on the bismuth(III) coordination geometry. In these compounds the Bi atom shows a tendency toward distorted octahedral coordination with some rather long Bi—Cl bonds, which is attributed to the aspherical distribution of the lone pair electrons at Bi(III).

The asymmetric unit of the title compound contains one fully protonated bis(3-azaniumylpropyl)azanium cation, two half of a [BiCl_6_]^3-^ anion and a neutral water molecule. A perspective view of the arrangement of these constituent entities is shown in Fig. 1 together with the atom numbering scheme. Two slightly distorted [BiCl_6_]^3-^ octahedra are located in special position on an inversion centre. The Bi–Cl bond lengths vary from 2.6817 (8) to 2.7209 (8) Å with an average bond lengths of 2.7014 (8) Å. These values are much shorter than the sum of the van der Waal radii of Bi and Cl (4.7 Å) according to Pauling (Pauling, 1960). In addition to the bond length differences, the Cl—Bi—Cl angles for the Cl atoms in *cis* position with respect to each other fall in the range of 85.80 (3)-94.20 (3)°. It should be mentioned that the Cl—Bi—Cl bond angles deviate substantially from 90° by 4.2° for Bi(1) and 3.1° for Bi(2). By taking into account the sixth-fold coordination of bismuth atoms, we have proceeded to calculate the bond-valence sum (BVS) of this metal using the parameters given by Brown (Brown *et al.*, 1985). The BVS calculation of the Bi1 and Bi2 ions gave respectively values of 3.23 and 3.38 valence units. These results confirm the presumed oxidation state of Bi(III). The distortion of the [BiCl_6_]^3-^ octahedral are correlated primary to the deformations resulting from the stereochemical activity of the Bi lone electron pair and secondary to deformations resulting from hydrogen bonding interactions The [BiCl_6_]^3-^ anions are connected through O—H···Cl hydrogen bonds (Table 1), so that [BiCl_6_(H_2_O)]_n_^3n-^ chains spread one-dimensionally parallel to the [0 -1 1] direction. The unit cell is crossed by two centrosymmetrical [BiCl_6_(H_2_O)]_n_^3n-^ chains with the (0 2 2) mid plane as shown in Fig. 2. The total negative charge (-3) on the framework is balanced by the presence of one independent fully protonated [NH_3_(CH_2_)_3_NH_2_(CH_2_)_3_NH_3_]^3+^ cation. The major contributions to the cohesion and the stability of the structure is provided by the presence of N—H···Cl and N—H···O hydrogen bonds linkages between the cations and the anionic chains belonging to adjacent (0 2 2) planes. All of these hydrogen bonds, N–H···Cl, N–H···O and O–H···Cl, give rise to a three-dimensional network in the structure (Fig. 3 and Table 1).

### 2. Experimental

Crystals of the title compound were obtained by dissolving in 100 ml of a solution of HCl (12*M*) a stoichiometric mixture of bismuth(III) oxide and bis(3-amino-propyl)amine (molar ratio 1:2). The resulting aqueous solution was then kept at room temperature. After several weeks of slow evaporation at room temperature, prismatic shaped monocrystals of the title compound were obtained. They were washed with diethyl ether and dried for 4 h over CaCl_2_.

### 3. Refinement

All H atoms belonging to the organic group cation were geometrically positioned and treated as riding on their parent atoms, with C—H = 0.97 Å and N—H = 0.89–0.90 Å and with *U*_iso_(H) = 1.2 *U*_eq_(C, N). The water H atoms were located in a difference Fourier map and refined using DFIX and DANG restraints. Their bond lengths were set to ideal values of 0.85 Å and finally they were refined using a riding model with *U*_iso_(H) = 1.5 *U*_eq_(O).

### Crystal data

**Table:**

(C_6_H_20_N_3_)[BiCl_6_]·H_2_O	*Z* = 2
*M**_r_* = 573.95	*F*(000) = 544
Triclinic, *P*1	Z=2
Hall symbol: -P 1	*D*_x_ = 2.165 Mg m^−^^3^*D*_m_ = 2.160 Mg m^−^^3^*D*_m_ measured by Flotation
*a* = 7.6891 (1) Å	Melting point: 430 K
*b* = 10.8642 (1) Å	Mo *K*α radiation, λ = 0.71073 Å
*c* = 11.9867 (1) Å	θ = 1.8–30.6°
α = 93.349 (1)°	µ = 10.91 mm^−^^1^
β = 108.509 (1)°	*T* = 296 K
γ = 109.387 (1)°	Prism, white
*V* = 880.54 (2) Å^3^	0.1 × 0.1 × 0.1 mm

### Data collection

**Table:**

Bruker APEXII CCD diffractometer	5325 independent reflections
Radiation source: fine-focus sealed tube	4009 reflections with *I* > 2σ(*I*)
Graphite monochromator	*R*_int_ = 0.025
ω scans	θ_max_ = 30.6°, θ_min_ = 1.8°
Absorption correction: multi-scan (*SADABS*; Bruker, 2006)	*h* = −10→10
*T*_min_ = 0.336, *T*_max_ = 0.349	*k* = −13→15
11734 measured reflections	*l* = −16→17

### Refinement

**Table:**

Refinement on *F*^2^	Secondary atom site location: difference Fourier map
Least-squares matrix: full	Hydrogen site location: inferred from neighbouring sites
*R*[*F*^2^ > 2σ(*F*^2^)] = 0.021	H atoms treated by a mixture of independent and constrained refinement
*wR*(*F*^2^) = 0.057	*w* = 1/[σ^2^(*F*_o_^2^) + (0.0323*P*)^2^] where *P* = (*F*_o_^2^ + 2*F*_c_^2^)/3
*S* = 0.92	(Δ/σ)_max_ = 0.001
5325 reflections	Δρ_max_ = 1.07 e Å^−^^3^
168 parameters	Δρ_min_ = −0.90 e Å^−^^3^
3 restraints	Extinction correction: *SHELXL97* (Sheldrick, 2008), Fc^*^=kFc[1+0.001xFc^2^λ^3^/sin(2θ)]^-1/4^
Primary atom site location: structure-invariant direct methods	Extinction coefficient: 0.0132 (4)

### Special details

**Table:**

Geometry. All e.s.d.'s (except the e.s.d. in the dihedral angle between two l.s. planes) are estimated using the full covariance matrix. The cell e.s.d.'s are taken into account individually in the estimation of e.s.d.'s in distances, angles and torsion angles; correlations between e.s.d.'s in cell parameters are only used when they are defined by crystal symmetry. An approximate (isotropic) treatment of cell e.s.d.'s is used for estimating e.s.d.'s involving l.s. planes.
Refinement. Refinement of *F*^2^ against ALL reflections. The weighted *R*-factor *wR* and goodness of fit *S* are based on *F*^2^, conventional *R*-factors *R* are based on *F*, with *F* set to zero for negative *F*^2^. The threshold expression of *F*^2^ > σ(*F*^2^) is used only for calculating *R*-factors(gt) *etc*. and is not relevant to the choice of reflections for refinement. *R*-factors based on *F*^2^ are statistically about twice as large as those based on *F*, and *R*- factors based on ALL data will be even larger.

### Fractional atomic coordinates and isotropic or equivalent isotropic displacement parameters (Å^2^)

**Table:**

	*x*	*y*	*z*	*U*_iso_*/*U*_eq_	
Bi1	0.5000	1.0000	0.5000	0.02401 (5)	
Bi2	0.5000	0.5000	0.0000	0.02614 (6)	
Cl1	0.32827 (12)	1.00845 (8)	0.66539 (8)	0.03793 (18)	
Cl2	0.19824 (12)	1.02948 (9)	0.32564 (8)	0.04104 (19)	
Cl3	0.31297 (13)	0.73550 (8)	0.42359 (10)	0.0451 (2)	
Cl4	0.43949 (12)	0.52414 (10)	0.20718 (8)	0.0461 (2)	
Cl5	0.12577 (12)	0.47555 (9)	−0.12177 (9)	0.0468 (2)	
Cl6	0.36405 (14)	0.23214 (9)	−0.03209 (11)	0.0523 (3)	
N1	1.1331 (4)	0.2550 (3)	0.6613 (2)	0.0383 (6)	
H1A	1.2346	0.3229	0.7126	0.057*	
H1B	1.1303	0.1800	0.6881	0.057*	
H1C	1.0211	0.2667	0.6541	0.057*	
C1	1.1551 (5)	0.2473 (3)	0.5432 (3)	0.0351 (7)	
H1E	1.0550	0.1664	0.4906	0.042*	
H1D	1.2834	0.2440	0.5526	0.042*	
C2	1.1357 (5)	0.3649 (3)	0.4873 (3)	0.0368 (7)	
H2E	1.2356	0.4457	0.5403	0.044*	
H2D	1.0074	0.3681	0.4782	0.044*	
C3	1.1583 (5)	0.3592 (3)	0.3652 (3)	0.0378 (8)	
H3E	1.1698	0.4437	0.3396	0.045*	
H3D	1.2784	0.3441	0.3720	0.045*	
N2	0.9871 (4)	0.2518 (2)	0.2741 (2)	0.0316 (6)	
H2A	0.9784	0.1738	0.2983	0.038*	
H2B	0.8761	0.2653	0.2702	0.038*	
C4	0.9967 (5)	0.2418 (3)	0.1523 (3)	0.0328 (7)	
H4E	1.1114	0.2220	0.1539	0.039*	
H4D	1.0091	0.3258	0.1254	0.039*	
C5	0.8107 (5)	0.1329 (3)	0.0668 (3)	0.0408 (8)	
H5E	0.6966	0.1481	0.0734	0.049*	
H5D	0.8065	0.0483	0.0903	0.049*	
C6	0.7976 (5)	0.1254 (4)	−0.0631 (3)	0.0472 (9)	
H6E	0.9246	0.1324	−0.0670	0.057*	
H6D	0.7013	0.0398	−0.1090	0.057*	
N3	0.7405 (4)	0.2322 (3)	−0.1174 (3)	0.0479 (8)	
H3A	0.6490	0.2444	−0.0925	0.072*	
H3B	0.6922	0.2089	−0.1968	0.072*	
H3C	0.8461	0.3071	−0.0955	0.072*	
OW	0.6532 (5)	0.2968 (4)	0.2841 (4)	0.0799 (11)	
H1W	0.576 (7)	0.300 (7)	0.217 (3)	0.15 (3)*	
H2W	0.597 (8)	0.252 (6)	0.328 (5)	0.16 (3)*	

### Atomic displacement parameters (Å^2^)

**Table:**

	*U*^11^	*U*^22^	*U*^33^	*U*^12^	*U*^13^	*U*^23^
Bi1	0.02304 (8)	0.02366 (8)	0.02407 (9)	0.00824 (5)	0.00767 (6)	0.00183 (6)
Bi2	0.02630 (8)	0.02556 (9)	0.02270 (9)	0.00658 (6)	0.00748 (6)	0.00122 (6)
Cl1	0.0370 (4)	0.0396 (4)	0.0405 (5)	0.0124 (3)	0.0195 (4)	0.0091 (3)
Cl2	0.0443 (4)	0.0527 (5)	0.0313 (4)	0.0292 (4)	0.0085 (3)	0.0076 (4)
Cl3	0.0399 (4)	0.0281 (4)	0.0656 (6)	0.0074 (3)	0.0238 (4)	−0.0002 (4)
Cl4	0.0397 (4)	0.0576 (5)	0.0310 (4)	0.0018 (4)	0.0186 (4)	−0.0010 (4)
Cl5	0.0317 (4)	0.0527 (5)	0.0532 (6)	0.0183 (4)	0.0086 (4)	0.0101 (4)
Cl6	0.0520 (5)	0.0267 (4)	0.0794 (8)	0.0109 (3)	0.0287 (5)	0.0106 (4)
N1	0.0349 (13)	0.0429 (16)	0.0286 (15)	0.0058 (11)	0.0097 (12)	0.0053 (12)
C1	0.0452 (17)	0.0347 (17)	0.0255 (16)	0.0160 (14)	0.0120 (14)	0.0030 (13)
C2	0.0467 (18)	0.0337 (17)	0.0257 (17)	0.0122 (14)	0.0109 (15)	0.0010 (13)
C3	0.0391 (16)	0.0335 (17)	0.0292 (18)	0.0019 (13)	0.0100 (14)	0.0037 (13)
N2	0.0374 (14)	0.0322 (15)	0.0219 (14)	0.0101 (11)	0.0094 (12)	0.0054 (11)
C4	0.0374 (15)	0.0355 (17)	0.0258 (16)	0.0138 (13)	0.0115 (13)	0.0053 (13)
C5	0.0507 (19)	0.0321 (17)	0.0313 (19)	0.0098 (14)	0.0104 (16)	0.0031 (14)
C6	0.0480 (19)	0.052 (2)	0.035 (2)	0.0197 (17)	0.0085 (17)	−0.0107 (17)
N3	0.0423 (16)	0.059 (2)	0.0293 (16)	0.0076 (14)	0.0075 (13)	0.0059 (14)
OW	0.089 (2)	0.114 (3)	0.088 (3)	0.069 (2)	0.055 (2)	0.067 (2)

### Geometric parameters (Å, º)

**Table:**

Bi1—Cl3	2.6976 (8)	C2—H2D	0.9700
Bi1—Cl3^i^	2.6976 (8)	C3—N2	1.485 (4)
Bi1—Cl2	2.7105 (8)	C3—H3E	0.9700
Bi1—Cl2^i^	2.7105 (8)	C3—H3D	0.9700
Bi1—Cl1^i^	2.7209 (8)	N2—C4	1.485 (4)
Bi1—Cl1	2.7209 (8)	N2—H2A	0.9000
Bi2—Cl4^ii^	2.6816 (8)	N2—H2B	0.9000
Bi2—Cl4	2.6817 (8)	C4—C5	1.517 (4)
Bi2—Cl5	2.6948 (8)	C4—H4E	0.9700
Bi2—Cl5^ii^	2.6948 (8)	C4—H4D	0.9700
Bi2—Cl6	2.7025 (9)	C5—C6	1.524 (5)
Bi2—Cl6^ii^	2.7025 (9)	C5—H5E	0.9700
N1—C1	1.478 (4)	C5—H5D	0.9700
N1—H1A	0.8900	C6—N3	1.485 (5)
N1—H1B	0.8900	C6—H6E	0.9700
N1—H1C	0.8900	C6—H6D	0.9700
C1—C2	1.506 (5)	N3—H3A	0.8900
C1—H1E	0.9700	N3—H3B	0.8900
C1—H1D	0.9700	N3—H3C	0.8900
C2—C3	1.528 (5)	OW—H1W	0.844 (19)
C2—H2E	0.9700	OW—H2W	0.857 (19)
			
Cl3—Bi1—Cl3^i^	180.0	C1—C2—H2E	109.1
Cl3—Bi1—Cl2	87.59 (3)	C3—C2—H2E	109.1
Cl3^i^—Bi1—Cl2	92.41 (3)	C1—C2—H2D	109.1
Cl3—Bi1—Cl2^i^	92.41 (3)	C3—C2—H2D	109.1
Cl3^i^—Bi1—Cl2^i^	87.59 (3)	H2E—C2—H2D	107.9
Cl2—Bi1—Cl2^i^	180.0	N2—C3—C2	111.4 (3)
Cl3—Bi1—Cl1^i^	85.80 (3)	N2—C3—H3E	109.4
Cl3^i^—Bi1—Cl1^i^	94.20 (3)	C2—C3—H3E	109.4
Cl2—Bi1—Cl1^i^	87.82 (3)	N2—C3—H3D	109.4
Cl2^i^—Bi1—Cl1^i^	92.18 (3)	C2—C3—H3D	109.4
Cl3—Bi1—Cl1	94.20 (3)	H3E—C3—H3D	108.0
Cl3^i^—Bi1—Cl1	85.80 (3)	C4—N2—C3	114.5 (3)
Cl2—Bi1—Cl1	92.18 (3)	C4—N2—H2A	108.6
Cl2^i^—Bi1—Cl1	87.82 (3)	C3—N2—H2A	108.6
Cl1^i^—Bi1—Cl1	180.0	C4—N2—H2B	108.6
Cl4^ii^—Bi2—Cl4	180.00 (4)	C3—N2—H2B	108.6
Cl4^ii^—Bi2—Cl5	89.74 (3)	H2A—N2—H2B	107.6
Cl4—Bi2—Cl5	90.26 (3)	N2—C4—C5	109.4 (3)
Cl4^ii^—Bi2—Cl5^ii^	90.26 (3)	N2—C4—H4E	109.8
Cl4—Bi2—Cl5^ii^	89.74 (3)	C5—C4—H4E	109.8
Cl5—Bi2—Cl5^ii^	180.00 (6)	N2—C4—H4D	109.8
Cl4^ii^—Bi2—Cl6	86.87 (3)	C5—C4—H4D	109.8
Cl4—Bi2—Cl6	93.13 (3)	H4E—C4—H4D	108.2
Cl5—Bi2—Cl6	87.00 (3)	C4—C5—C6	113.0 (3)
Cl5^ii^—Bi2—Cl6	93.00 (3)	C4—C5—H5E	109.0
Cl4^ii^—Bi2—Cl6^ii^	93.13 (3)	C6—C5—H5E	109.0
Cl4—Bi2—Cl6^ii^	86.87 (3)	C4—C5—H5D	109.0
Cl5—Bi2—Cl6^ii^	93.00 (3)	C6—C5—H5D	109.0
Cl5^ii^—Bi2—Cl6^ii^	87.00 (3)	H5E—C5—H5D	107.8
Cl6—Bi2—Cl6^ii^	180.00 (6)	N3—C6—C5	112.2 (3)
C1—N1—H1A	109.5	N3—C6—H6E	109.2
C1—N1—H1B	109.5	C5—C6—H6E	109.2
H1A—N1—H1B	109.5	N3—C6—H6D	109.2
C1—N1—H1C	109.5	C5—C6—H6D	109.2
H1A—N1—H1C	109.5	H6E—C6—H6D	107.9
H1B—N1—H1C	109.5	C6—N3—H3A	109.5
N1—C1—C2	111.4 (3)	C6—N3—H3B	109.5
N1—C1—H1E	109.4	H3A—N3—H3B	109.5
C2—C1—H1E	109.4	C6—N3—H3C	109.5
N1—C1—H1D	109.4	H3A—N3—H3C	109.5
C2—C1—H1D	109.4	H3B—N3—H3C	109.5
H1E—C1—H1D	108.0	H1W—OW—H2W	115 (3)
C1—C2—C3	112.3 (3)		
			
N1—C1—C2—C3	179.9 (3)	C3—N2—C4—C5	−177.7 (3)
C1—C2—C3—N2	69.7 (4)	N2—C4—C5—C6	173.8 (3)
C2—C3—N2—C4	178.8 (3)	C4—C5—C6—N3	−76.5 (4)

Symmetry codes: (i) −*x*+1, −*y*+2, −*z*+1; (ii) −*x*+1, −*y*+1, −*z*.

### Hydrogen-bond geometry (Å, º)

**Table:**

*D*—H···*A*	*D*—H	H···*A*	*D*···*A*	*D*—H···*A*
N1—H1*A*···Cl4^iii^	0.89	2.34	3.174 (3)	156
N1—H1*B*···Cl2^iv^	0.89	2.73	3.339 (3)	127
N1—H1*B*···Cl1^v^	0.89	2.82	3.474 (3)	132
N1—H1*C*···Cl3^iv^	0.89	2.43	3.293 (3)	163
N2—H2*B*···O*W*	0.90	1.91	2.804 (4)	173
N2—H2*A*···Cl2^v^	0.90	2.63	3.316 (3)	134
N2—H2*A*···Cl1^iv^	0.90	2.71	3.347 (3)	129
N3—H3*A*···Cl6	0.89	2.48	3.362 (3)	169
N3—H3*B*···Cl1^vi^	0.89	2.81	3.412 (3)	126
N3—H3*B*···Cl3^ii^	0.89	2.81	3.612 (3)	151
N3—H3*C*···Cl5^vii^	0.89	2.44	3.269 (3)	155
O*W*—H1*W*···Cl6	0.84 (2)	2.83 (3)	3.620 (4)	157 (6)
O*W*—H2*W*···Cl3^iv^	0.86 (2)	2.82 (4)	3.478 (4)	135 (5)

Symmetry codes: (ii) −*x*+1, −*y*+1, −*z*; (iii) −*x*+2, −*y*+1, −*z*+1; (iv) −*x*+1, −*y*+1, −*z*+1; (v) *x*+1, *y*−1, *z*; (vi) *x*, *y*−1, *z*−1; (vii) *x*+1, *y*, *z*.

## References

[bb1] Brown, I. D. & Altermatt, D. (1985). *Acta Cryst.* B**41**, 244–247.

[bb2] Bruker (2006). *APEX2*, *SAINT* and *SADABS* Bruker AXS Inc., Madison, Wisconsin, USA.

[bb3] Chaabouni, S., Kamoun, S. & Jaud, J. (1998). *J. Chem. Crystallogr.* **28**, 209–212.

[bb4] Farrugia, L. J. (2012). *J. Appl. Cryst.* **45**, 849–854.

[bb5] Fu, Y.-L., Xu, Z.-W., Ren, J.-L. & Ng, S. W. (2005). *Acta Cryst.* E**61**, m1717–m1718.

[bb6] Macrae, C. F., Bruno, I. J., Chisholm, J. A., Edgington, P. R., McCabe, P., Pidcock, E., Rodriguez-Monge, L., Taylor, R., van de Streek, J. & Wood, P. A. (2008). *J. Appl. Cryst.* **41**, 466–470.

[bb7] Ouasri, A., Rhandour, A., Saadi, M. & El Ammari, L. (2013). *Acta Cryst.* E**69**, m437. 10.1107/S1600536813018102 PMC3793680 24109267

[bb8] Pauling, L. (1960). *The Nature of the Chemical Bond* p. 260. Ithaca: Cornell University Press.

[bb9] Rhandour, A., Ouasri, A., Roussel, P. & Mazzah, A. (2011). *J. Mol. Struct.* **990**, 95–101.

[bb10] Sheldrick, G. M. (2008). *Acta Cryst.* A**64**, 112–122. 10.1107/S0108767307043930 18156677

[bb11] Westrip, S. P. (2010). *J. Appl. Cryst.* **43**, 920–925.

